# Whole-transcriptome insights into follicle selection: deciphering key regulatory networks in Luxi gamecock

**DOI:** 10.3389/fgene.2025.1620058

**Published:** 2025-08-06

**Authors:** Yiya Wang

**Affiliations:** School of Life Sciences, Qilu Normal University, Jinan, China

**Keywords:** Luxi gamecock, follicle selection, RNA-seq, differential expression RNAs, ceRNA network

## Abstract

**Background:**

Luxi gamecock is a native Chinese breed recognized for its substantial body size, well-developed musculature, and aggressive behavior. Despite these advantageous traits, the breed’s egg production rate remains relatively low, insufficient to meet market demands. Follicle selection plays a crucial role in determining the egg-laying performance of hens, yet research on follicle selection in Luxi gamecock is limited. In this study, RNA sequencing was performed on small yellow follicles (SYFs) and large yellow follicles (LYFs) from Luxi gamecock to identify RNA transcript expression, and subsequent RNA networks were constructed.

**Methods:**

SYFs and LYFs were collected from 15 Luxi gamecocks and divided randomly into three biological groups. RNA was isolated to profile the expression of mRNA, lncRNA, circRNA, and miRNA. The results were validated using qRT-PCR. Functional analysis, including GO and KEGG, was conducted. Competitive endogenous RNA (ceRNA) networks were also constructed.

**Results:**

A total of 1,113 mRNAs, 245 lncRNAs, 264 circRNAs, and 90 miRNAs were differentially expressed between SYFs and LYFs. qRT-PCR validation showed high consistency with the RNA-seq results. Functional enrichment indicated that these differentially expressed RNAs are associated with critical biological processes and involved in several key signaling pathways. To investigate the potential interactions among circRNAs, lncRNAs, and miRNAs, ceRNA networks were constructed.

**Conclusion:**

This study provides a detailed characterization of the transcriptomes in SYFs and LYFs of Luxi gamecock through RNA sequencing. The functional analysis revealed that many RNAs may contribute to follicle selection. Furthermore, ceRNA networks were built to better understand the molecular mechanisms behind follicle selection. These findings shed light on the potential regulatory roles of various RNA molecules in the follicle selection of Luxi gamecock, and also uncover the interactions among them, laying a foundation for improving the breed’s egg-laying performance.

## 1 Introduction

**FIGURE 1:**
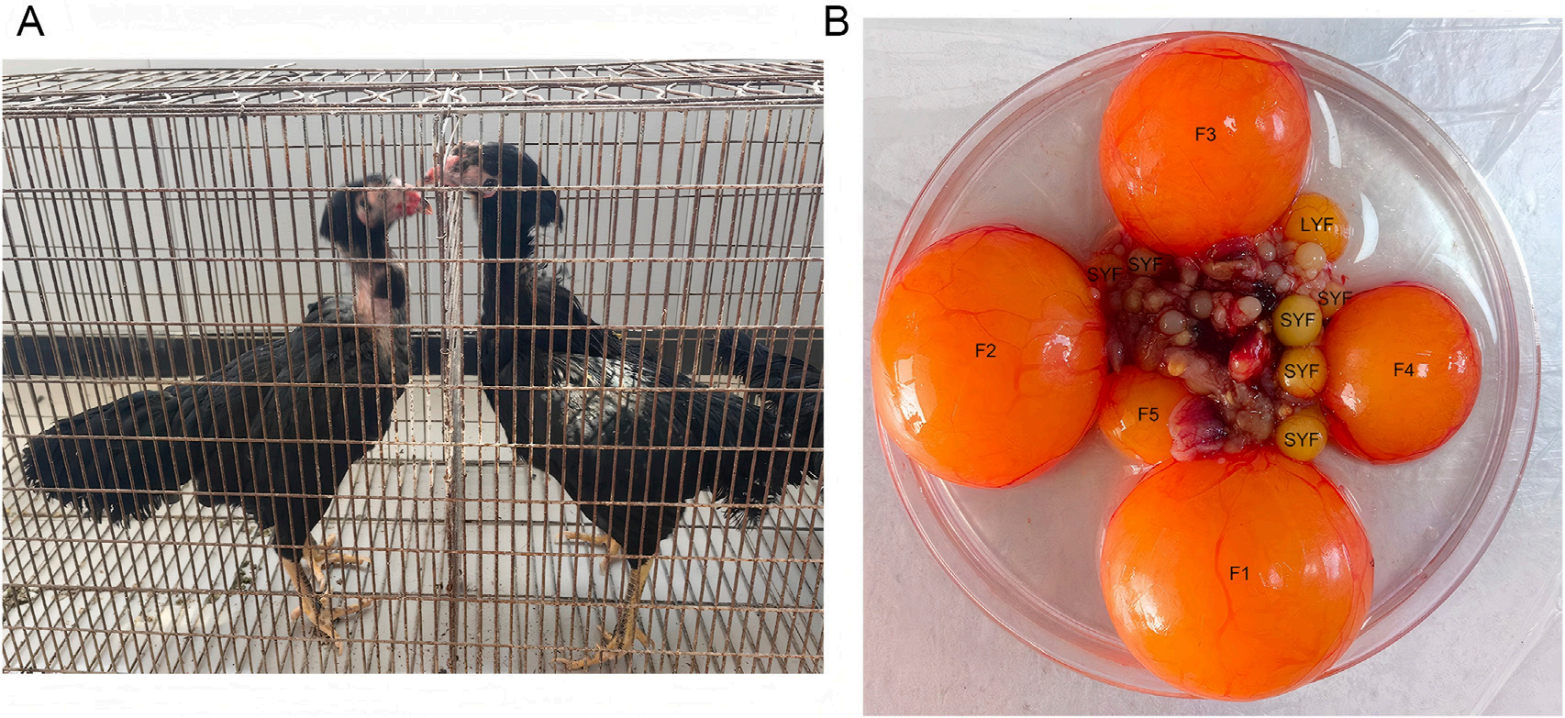
**(A)** Hens and **(B)** follicle grades of Luxi gamecock.

Chinese gamecocks have been selectively bred for over 2500 years. These birds are highly valued as a precious poultry resource with substantial potential in the agricultural sector (Liu et al., 2006). There are numerous indigenous gamecock breeds throughout the country, including the Luxi gamecock, Henan gamecock, Tulufan gamecock, Xishuangbannan gamecock, and Zhangxi gamecock. These local breeds represent an untapped resource with significant genetic diversity, urgently in need of further development and utilization. Luxi gamecock, in particular, stands out due to its robust and muscular build, with a larger and more imposing stature compared to many other breeds. This breed is known for its excellent physique, with adult hens typically weighing between 2.8 and 3.1 kg (Figure 1A). The Luxi gamecock breed faces challenges regarding its reproductive performance. Luxi gamecocks begin egg production relatively late, usually between 200 and 240 days of age, and the hens typically produce around 60 to 100 eggs annually. The low production rate limits the commercial viability of this breed and is also unfavorable for the conservation and development of its germplasm resources.

In the ovaries of sexually mature hens are follicles of varying sizes and stages of development, following a highly structured and hierarchical system of growth and ovulation. These follicles can be classified into pre-hierarchical types based on their size, including SWFs (small white follicles) that measure less than 3 mm, LWFs (large white follicles) ranging from 3 to 5 mm, and SYFs that measure between 6 and 8 mm. In addition to these, there are also follicle grades, such as the F6 to F1 range, with LYFs measuring between 9 and 12 mm in diameter (Johnson, 2015) (Figure 1B). As the hen reaches its peak egg-laying period, one of the SYFs is selected daily to enter the hierarchical follicle system. This follicle then begins rapid growth and development, eventually proceeding to ovulation. This selective process, known as follicle selection (Johnson and Woods, 2009), plays a pivotal role in regulating reproductive performance and fecundity in hens (Johnson et al., 2015). Although extensive research has been carried out to understand the mechanisms involved in follicle selection in chickens (Guo et al., 2019; Zhou et al., 2020; Nie et al., 2022; Wang et al., 2017), the precise molecular mechanisms remain largely undefined. Furthermore, there is currently no research on the molecular mechanism of follicle selection in Luxi gamecock. Exploring the molecular events during the follicle selection can help screen potential molecular markers. These markers may serve as genetic indicators for marker-assisted selection or provide information for screening key functional genes and studying expression regulatory mechanisms during follicle selection, thus providing a scientific basis for improving the egg-laying performance of Luxi gamecock through deciphering reproductive regulatory networks.

Non-coding RNAs (ncRNAs) represent a diverse category of functionally active transcripts that lack protein-coding potential. This group encompasses various subtypes, including microRNAs (miRNAs), transfer RNAs, ribosomal RNAs, small interfering RNAs, and long noncoding RNAs (lncRNAs) (Gomes et al., 2013). Although they do not encode proteins, these RNA molecules play crucial roles in regulating the expression of protein-coding genes and modulating various cellular and physiological functions. miRNAs, characterized by their 21–24 nucleotide lengths, exert broad-spectrum regulatory effects on biological pathways ranging from cellular proliferation to programmed cell death. Multiple miRNAs are involved in key processes in poultry, including follicle selection, ovarian development, steroid hormone synthesis, and the regulation of egg-laying performance (Wang et al., 2021; Xu et al., 2024; He et al., 2022; Wei et al., 2022). LncRNAs are defined as transcripts exceeding 200 nucleotides in length (St. Laurent et al., 2015). Certain lncRNAs containing complementary binding sites for miRNAs can function as ceRNAs, competitively binding miRNAs to derepress downstream target genes (Bridges et al., 2021). Moreover, lncRNA expression displays remarkable spatiotemporal specificity, exhibiting dynamic stage-dependent variations within identical tissues that underscore their sophisticated regulatory nature (Dinger et al., 2008). Several studies have demonstrated the involvement of lncRNAs in regulating follicle development in chickens (Peng et al., 2019; Zhang et al., 2023), and further investigations have explored how lncRNA expression is linked to follicle selection (Zhong et al., 2021).

Circular RNAs (circRNAs) are RNA molecules that are characterized by their closed-loop structure, which results from a non-canonical reverse splicing process. These molecules are expressed in a highly regulated, tissue-specific, and time-dependent manner across various cell types, contributing to a wide array of molecular and biological activities (Xu et al., 2017). CircRNAs can act as sponges for miRNAs, thereby modulating miRNA availability and function (Hansen et al., 2013). Recent studies have provided evidence that circRNAs are involved in the intricate regulation of ovarian functions, playing pivotal roles in maintaining reproductive health and proper ovarian performance (Zhou et al., 2020).

ceRNA represents a complex network of interactions between different RNA molecules, including both protein-coding messenger RNAs and ncRNAs, such as lncRNAs and circRNAs (Ala, 2020). These RNA species function as either ceRNAs or as natural miRNA sponges, engaging in competitive binding to shared MREs (miRNA response elements) on target genes. This competition regulates gene expression by modulating the availability of miRNAs, ultimately influencing the transcriptional activity of various genes (Salmena et al., 2011). ceRNAs play vital roles in numerous biological processes, including gene regulation, cellular differentiation, and tissue development, as well as in various pathophysiological conditions (Singh et al., 2022). While the ceRNA mechanism has gained broad recognition as a key regulatory pathway in gene expression, there is limited research on the role of ceRNAs in specific processes such as chicken follicle selection, particularly in the case of Luxi gamecock, where such studies remain scarce.

To gain further insight into the molecular events associated with follicle selection in Luxi gamecock, transcriptomic profiling of Luxi gamecock follicles (SYFs and LYFs) was conducted to delineate transcriptional signatures across four RNA categories: mRNAs, lncRNAs, circRNAs, and miRNAs. This comparative analysis sought to detect differentially expressed transcripts associated with follicle selection processes. A ceRNA network associated with follicle selection was constructed to highlight key genes and related signaling pathways involved in this process. Overall, this investigation provides a foundation for future studies of these genes and their functions in regulating follicle selection, and these genes can be used as potential molecular markers for further research of Luxi gamecock. These findings can also provide ideas for future research on increasing the egg production of Luxi gamecocks.

## 2 Materials and methods

### 2.1 Animals and sample collection

The Luxi gamecocks used in this investigation were provided by the Luxi Gamecock Breed Resource Conservation Farm (Shandong, China). These chickens were housed in individual cages, with a controlled light schedule providing 14 h of light each day. The hens were granted free access to both water and feed. A total of 15 hens, each 35 weeks old, were randomly chosen for the study. These hens were slaughtered by cervical dislocation and dissected to collect follicles of varying sizes that did not contain yolk. Following a previously reported sampling strategy (Chen et al., 2020), 15 Luxi gamecocks were randomly allocated into three independent biological replicates (n = 5 hens/replicate). Each replicate consisted of pooled follicle samples from five distinct hens, with SYFs and LYFs collected from the same individuals (i.e., SYF-1/LYF-1, SYF-2/LYF-2 and SYF-3/LYF-3 each corresponded to a unique set of five hens, with no overlap between replicates). The SYFs and LYFs, with diameters of 6–8 mm and 9–12 mm, respectively, were collected and kept separately. Follicles were frozen immediately in liquid nitrogen and preserved at −80°C. All the animal experiments were approved by the Institutional Animal Care and Use Committee of Qilu Normal University (Approval No.: xsllsc2023-03).

### 2.2 RNA extraction, library construction, and sequencing

After sampling, total RNA extraction was performed using TRIzol reagent (Invitrogen, Carlsbad, CA, United States) following the manufacturer’s recommended procedures. The extractions carried out within 1 week to maintain optimal nucleic acid purity. The integrity of the extracted RNA was initially evaluated with an Agilent 2100 Bioanalyzer (Agilent Technologies, Palo Alto, CA, United States). RNase-free agarose gel electrophoresis was employed as a secondary method to verify the absence of degradation. cDNA libraries were constructed, followed by sequencing on the Illumina NovaSeq 6000, facilitated by Gene Denovo Biotechnology Co., Ltd (Guangzhou, China). This RNA sequencing study incorporated three independent biological replicates to ensure statistical robustness.

### 2.3 Bioinformatics analysis

Raw sequence data were filtered using fastp (v0.18.0) (Chen S. et al., 2018), aligned to the chicken genome with HISAT v2.2.4 (Ensembl_release 106) (Kim et al., 2015), and assembled with StringTie v1.3.1 (Pertea et al., 2015; Pertea et al., 2016). Protein-coding potential was assessed using CNCI (v2) (Sun et al., 2013), CPC (v0.9-r2) (Kong et al., 2007), and FEELNC (v0.2) (Wucher et al., 2017). The results from these tools were intersected to identify lncRNAs. These lncRNAs were then classified into five classes based on their proximity and relationship to protein-coding genes. The expression levels and variability of the RNAs were quantified using the FPKM method, with calculations performed using the RSEM software (Li and Dewey, 2011). The correlation between biological replicates was evaluated using R, which provided insight into the repeatability of the samples. Principal Component Analysis (PCA) was conducted using the gmodels package in R (http://www.rproject.org/) to investigate the relationships between the different samples. Differential expression of mRNAs, circRNAs, and lncRNAs was determined using the DESeq2 software (Love et al., 2014), with the filtering criteria set to fold change ≥ 2 and FDR < 0.05. Functional enrichment analyses, including Gene Ontology (GO) (Ashburner et al., 2000) and Kyoto Encyclopedia of Genes and Genomes (KEGG) (Kanehisa, 2000), were performed on the differentially expressed mRNAs, circRNAs, and lncRNAs. Gene Set Enrichment Analysis (GSEA) was performed using the GSEA software and MSigDB (Subramanian et al., 2005). RNAplex (v0.2) (Shen et al., 2014) was used to predict interactions. For circRNAs annotated in circBase, interactions with miRNAs were predicted using StarBase (v2.0). The target relationships for novel circRNAs were predicted using Mireap, Miranda (v3.3a), and TargetScan (v7.0) (Qiao et al., 2016). Furthermore, miRTarBase (v6.1) (Hsu et al., 2011) was employed to predict the mRNAs targeted by miRNA sponges formed by circRNAs.

Six cDNA libraries were prepared for small RNA sequencing. Low-quality reads were removed, and clean reads were aligned with reads from GeneBank, Rfam, and miRBase databases. Novel miRNA candidates were identified using the software mirdeep2. miRNA expression levels were calculated and normalized to transcripts per million (TPM). The criteria for filtering the differentially expressed (DE) miRNAs were fold change ≥ 2 and *p*-value < 0.05. Miranda (v3.3a) and TargetScan (v7.0) were used to explore potential miRNA target genes. For functional and biological analysis, GO terms and KEGG pathways were employed to gain a deeper understanding of the roles these miRNAs might play in various cellular processes.

### 2.4 Analysis and construction of ceRNA networks

Differential expression was identified using edgeR (Robinson et al., 2010). Three distinct tools were employed to predict the target genes of miRNAs: mireap, miRanda, and TargetScan. The expression correlations between miRNAs and their respective target genes were analyzed using the Spearman Rank correlation coefficient (SCC) (Xu et al., 2016), and pairs that had an SCC value less than −0.7 were selected for further investigation as potential miRNA-mRNA, miRNA-lncRNA, or miRNA-circRNA interactions. Subsequently, Pearson correlation coefficient (PCC) was used to calculate the relationship between the ceRNA pairs identified in the previous step. Those pairs with a PCC greater than 0.9 were considered as potential candidate ceRNA pairs for further analysis. ceRNA pairs were identified using hypergeometric test (*p* < 0.05). Based on the data and ceRNA hypothesis, a comprehensive ceRNA network was constructed, which was then visually represented using Cytoscape software (v3.6.0). ceRNA connectivity analysis was conducted to pinpoint hub genes, which play central roles in the network. For functional insights, GO and KEGG pathway analyses were applied to the mRNAs within the ceRNA network using Cytoscape, helping to explore the biological processes and pathways these mRNAs are involved in.

### 2.5 Quantitative reverse transcription PCR (qRT-PCR)

To validate RNA-seq findings, 32 DE mRNAs and 16 DE miRNAs in various follicle types were analyzed using qRT-PCR. In order to improve the statistical power and enhance the credibility of the results, 5 samples in each of the SYF and LYF groups were tested, including the three samples for sequencing. Primers were designed using Primer Premier 6.0 (Supplementary Table S1). cDNA was synthesized using Evo M-MLV RT Mix Kit with gDNA Clean for qPCR Ver.2 (Accurate Biology, Beijing, China) (mRNA) and miRNA first Strand cDNA Synthesis Kit (by tailing A) (Vazyme, Nanjing, China) (miRNA). qRT-PCR was conducted using SYBR Green Premix Pro Taq HS qPCR Kit (Accurate Biology, Beijing, China) or ChamQ Universal SYBR qPCR Master Mix (Vazyme, Nanjing, China). The 2^−△△CT^ method was used for gene expression calculation (Schmittgen and Livak, 2008), with ACTB and 18s as reference controls.

### 2.6 Statistical analyses

All data are presented as the mean ± standard error of mean (SEM). After tests for normal distribution and homogeneity of variance, differences between groups were determined using an independent samples t-test, performed with SPSS 26.0 statistical software (SPSS Inc., Chicago, IL, United States). Differences between groups were considered significant at *p* < 0.05.

## 3 Results

### 3.1 RNA-seq overview

**FIGURE 2:**
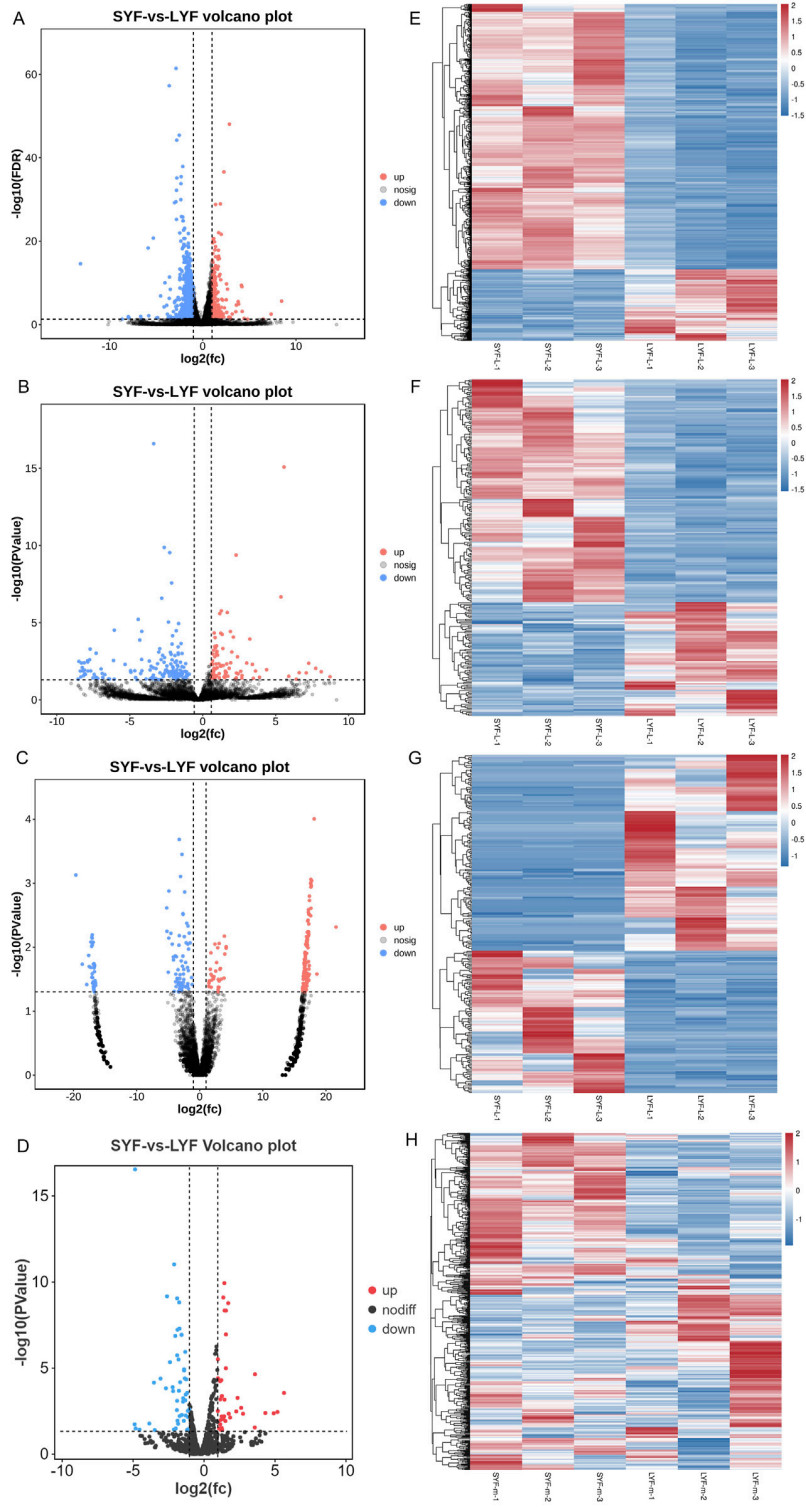
Heatmap and volcano plot representations of differentially expressed RNAs. The heatmaps display changes in expression for **(A)** mRNAs, **(B)** lncRNAs, **(C)** circRNAs, and **(D)** miRNAs. Volcano plots depict differential expression for **(E)** mRNAs, **(F)** lncRNAs, **(G)** circRNAs, and **(H)** miRNAs. In both visualizations, red indicates genes with higher expression levels, while blue represents those with lower expression levels.

In this study, SYF and LYF samples were collected from Luxi gamecocks to investigate the respective transcriptomes of these follicle types. Both raw and mapped read data are provided in Supplementary Table S2. A total of 17,187 mRNAs, 9,458 lncRNAs, 17,809 circRNAs, and 992 miRNAs identified in the follicles are summarized in Supplementary Table S3. To assess the consistency and reproducibility of the data, PCA was conducted and Pearson correlation coefficients were calculated between samples. This analysis revealed a high degree of correlation among samples in each group (Supplementary Figure S1). Subsequently, differentially expressed genes (DEGs) were identified between SYFs and LYFs. The analysis uncovered 1,113 mRNAs, 245 lncRNAs, 264 circRNAs, and 90 miRNAs that exhibited significant differential expression between the two follicle types (Table 1; Supplementary Table S4). Volcano plots illustrating the DE mRNAs, lncRNAs, circRNAs, and miRNAs are shown in Figures 2A–D, and the results of the hierarchical clustering analysis are depicted in Figures 2E–H. These findings highlight the distinct expression patterns in SYFs and LYFs.

**TABLE 1:** Summary of the number of differentially expressed mRNAs, lncRNAs, circRNAs, and miRNAs that exhibit statistically significant changes between the SYF and LYF sample groups.

RNA	Upregulated	Downregulated	Total
mRNA	236	877	1113
lncRNA	83	162	245
circRNA	153	111	264
miRNA	40	50	90

### 3.2 Validation of RNA-Seq results using qRT-PCR

**FIGURE 3:**
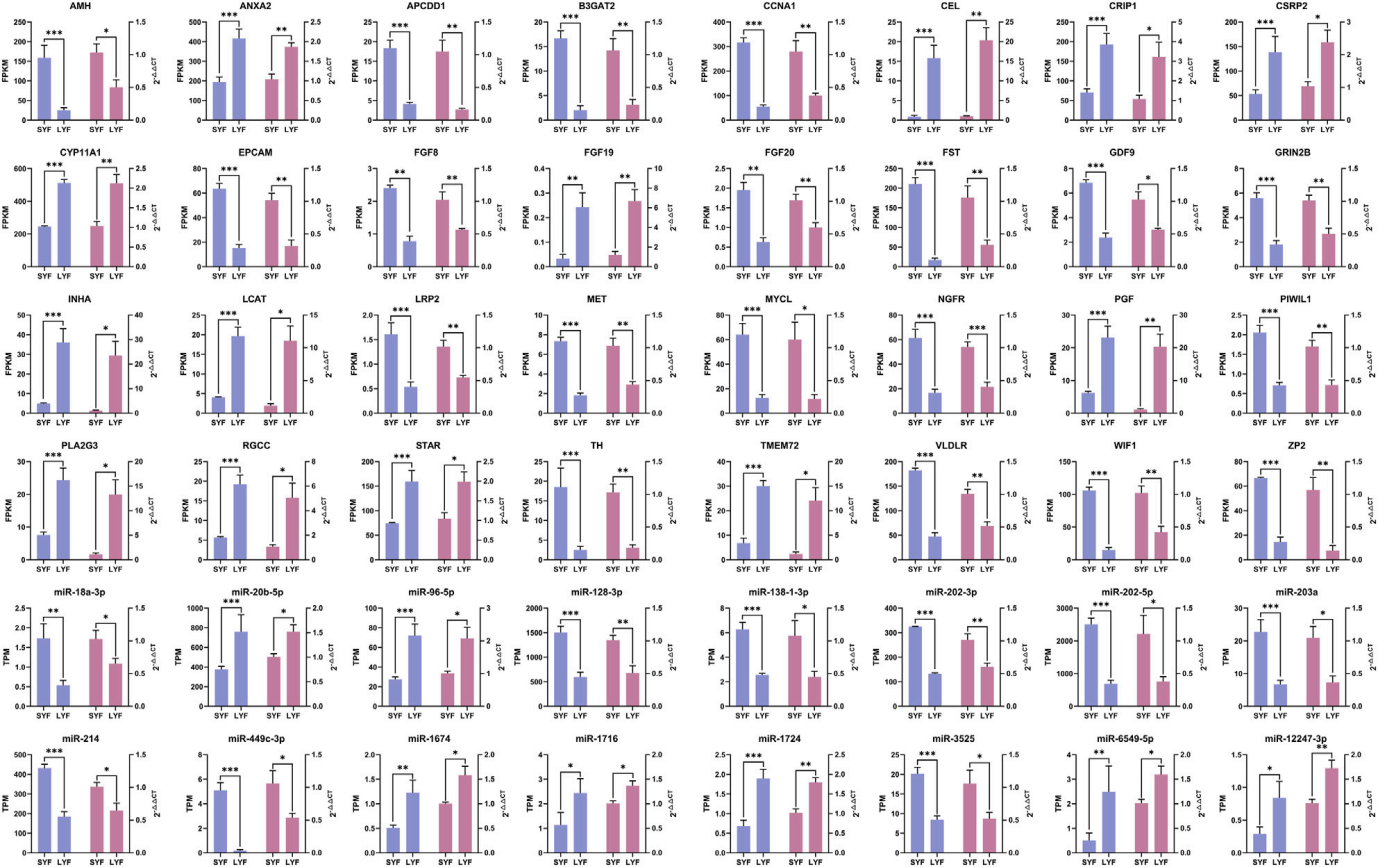
Validation of mRNA and miRNA expression levels using qRT-PCR based on RNA-seq data. Gene expression was normalized using ACTB and 18S rRNA as references. Results are presented as mean ± SEM (n = 5). The blue bars correspond to RNA-seq results, while the pink bars represent data from qRT-PCR. Statistical significance is denoted as *p < 0.05, **p < 0.01, and ***p < 0.001.

To validate the RNA-seq findings, a selection of 32 mRNAs and 16 miRNAs was analyzed using qRT-PCR (Figure 3). The expression profiles of these chosen mRNAs and miRNAs were consistent with the RNA-seq data, showing congruent trends across both methodologies. These findings further confirmed the accuracy and reliability of the sequencing results, demonstrating a clear match in expression patterns between the two approaches.

### 3.3 Function analysis

#### 3.3.1 DE mRNAs

**FIGURE 4:**
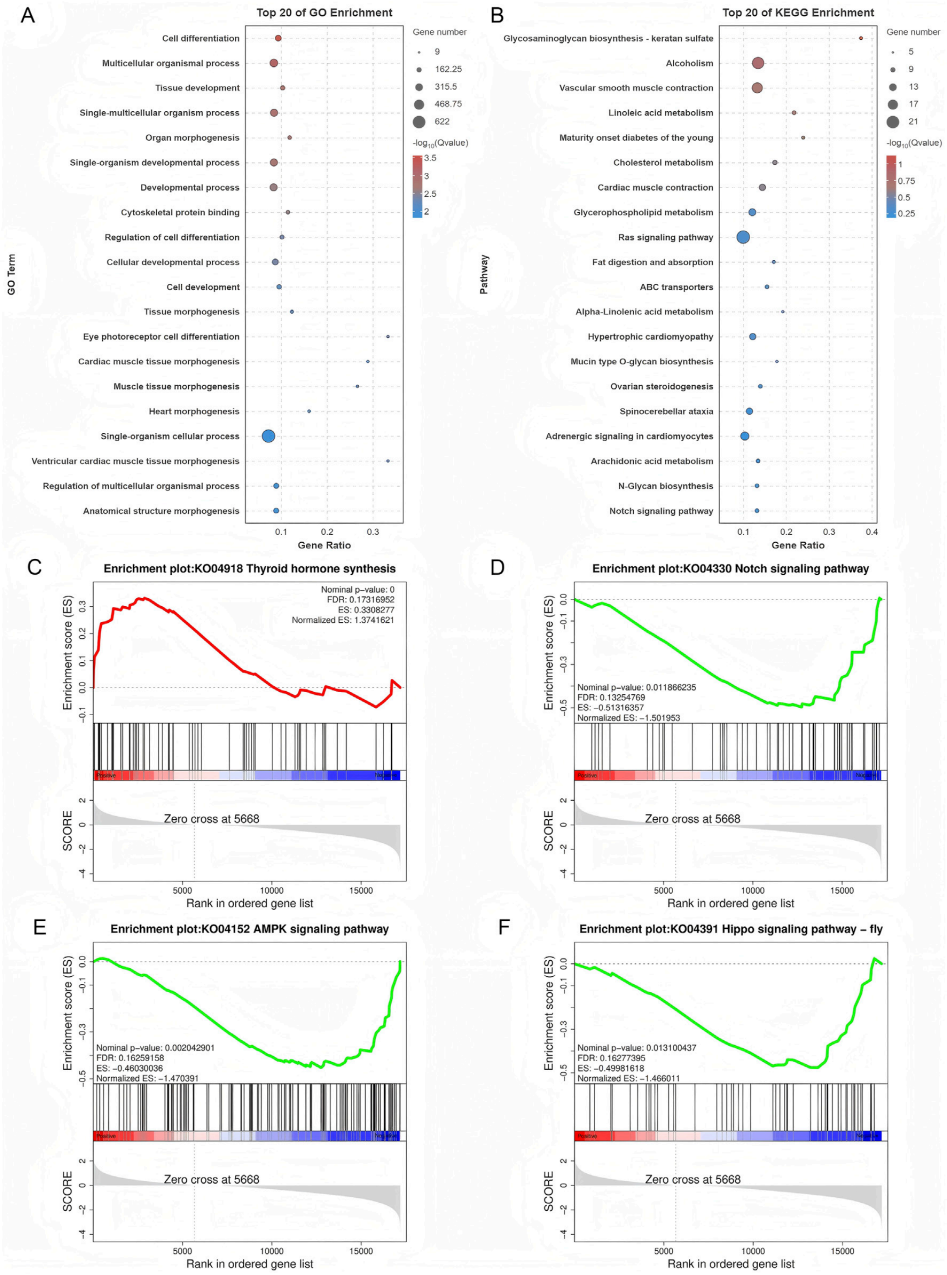
Functional characterization of differentially expressed mRNAs. **(A)** GO analysis of the differentially expressed mRNAs. **(B)** KEGG pathway analysis of the differentially expressed mRNAs. Enrichment plots for the following pathways: **(C)** Thyroid hormone synthesis, **(D)** Notch signaling pathway, **(E)** AMPK signaling pathway, and **(F)** Hippo signaling pathway.

GO and KEGG pathway analyses of DE mRNAs revealed 787 enriched GO pathways (*p* < 0.05) (Supplementary Table S5), including 619 biological processes, 56 cellular components, and 112 molecular functions, and 20 enriched KEGG pathways (*p* < 0.05), including Ras signaling, ovarian steroidogenesis, and Notch signaling. The top 20 GO and KEGG enrichment pathways are shown in Figures 4A,B. GSEA was conducted to further explore the gene sets that showed differential expression between SYFs and LYFs. The results showed positive enrichment in pathways associated with thyroid hormone synthesis, while several pathways, such as those in the Notch, AMPK, and Hippo signaling pathways, were negatively enriched in LYFs compared to SYFs (Figures 4C–F). The complete list of significantly enriched pathways identified using GSEA is provided in Supplementary Table S5 (|NES| > 1, *p* < 0.05). These findings suggest that the DE mRNAs may regulate key processes involved in follicle selection.

#### 3.3.2 DE lncRNAs

**FIGURE 5:**
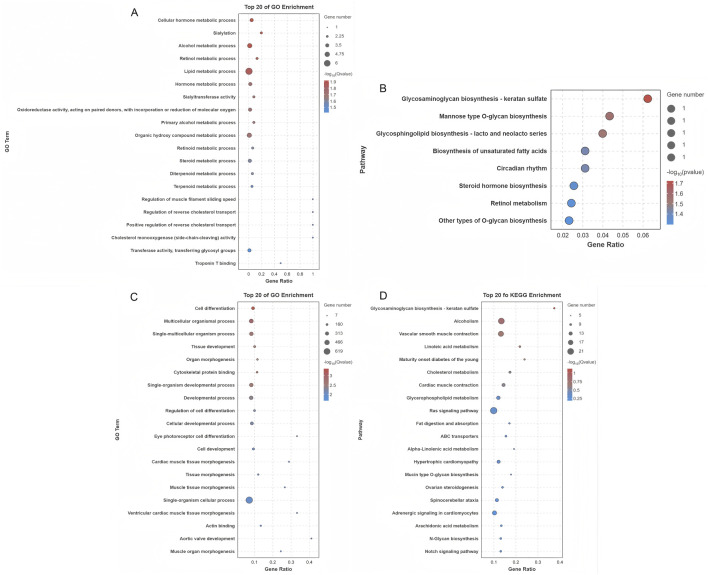
Functional characterization of targets of differentially expressed lncRNAs. **(A)** GO functional enrichment analysis of cis-regulatory targets of lncRNAs. **(B)** KEGG pathway analysis of cis-regulatory lncRNA targets. **(C)** GO functional enrichment analysis of trans-regulatory lncRNA targets. **(D)** KEGG pathway analysis of trans-regulatory lncRNA targets.

Cis-regulatory target genes of DE lncRNAs were analyzed, with a particular focus on protein-coding genes located within 10 kb. This analysis identified 21 DE lncRNAs corresponding to 19 protein-coding genes in close proximity (Supplementary Table S6). GO analysis of these target genes revealed significant enrichment in 134 terms (*p* < 0.05), predominantly associated with various metabolic processes such as cellular hormone metabolism, organic hydroxy compound metabolism, and steroid metabolism, all of which are essential for maintaining cellular homeostasis (Figure 5A; Supplementary Table S6). Additionally, KEGG pathway analysis revealed that these cis-regulated genes were significantly enriched in eight major pathways, with steroid hormone biosynthesis being one of the prominent pathways identified (Figure 5B). These results suggest that DE lncRNAs may regulate processes associated with follicle selection through cis regulation of neighboring target genes. The predicted trans-regulatory roles of DE lncRNAs were also explored based on their expression correlation coefficients (|Pearson r| ≥ 0.95), leading to the identification of 28,389 predicted interactions between 242 DE lncRNAs and 1,107 protein-coding genes (Supplementary Table S6). Functional enrichment analysis of these trans-regulated genes showed significant association with 786 GO terms, with key processes such as cell differentiation, tissue development, and cellular developmental processes being strongly represented (Figure 5C; Supplementary Table S6). In parallel, KEGG pathway analysis indicated that these trans-regulatory targets were enriched in 20 key pathways, including the Ras signaling pathway, ovarian steroidogenesis, and Notch signaling pathway (Figure 5D). This extensive functional annotation suggests that DE lncRNAs may exert their regulatory influence on follicle selection through the trans-regulation of a wide spectrum of target genes.

#### 3.3.3 DE circRNAs

**FIGURE 6:**
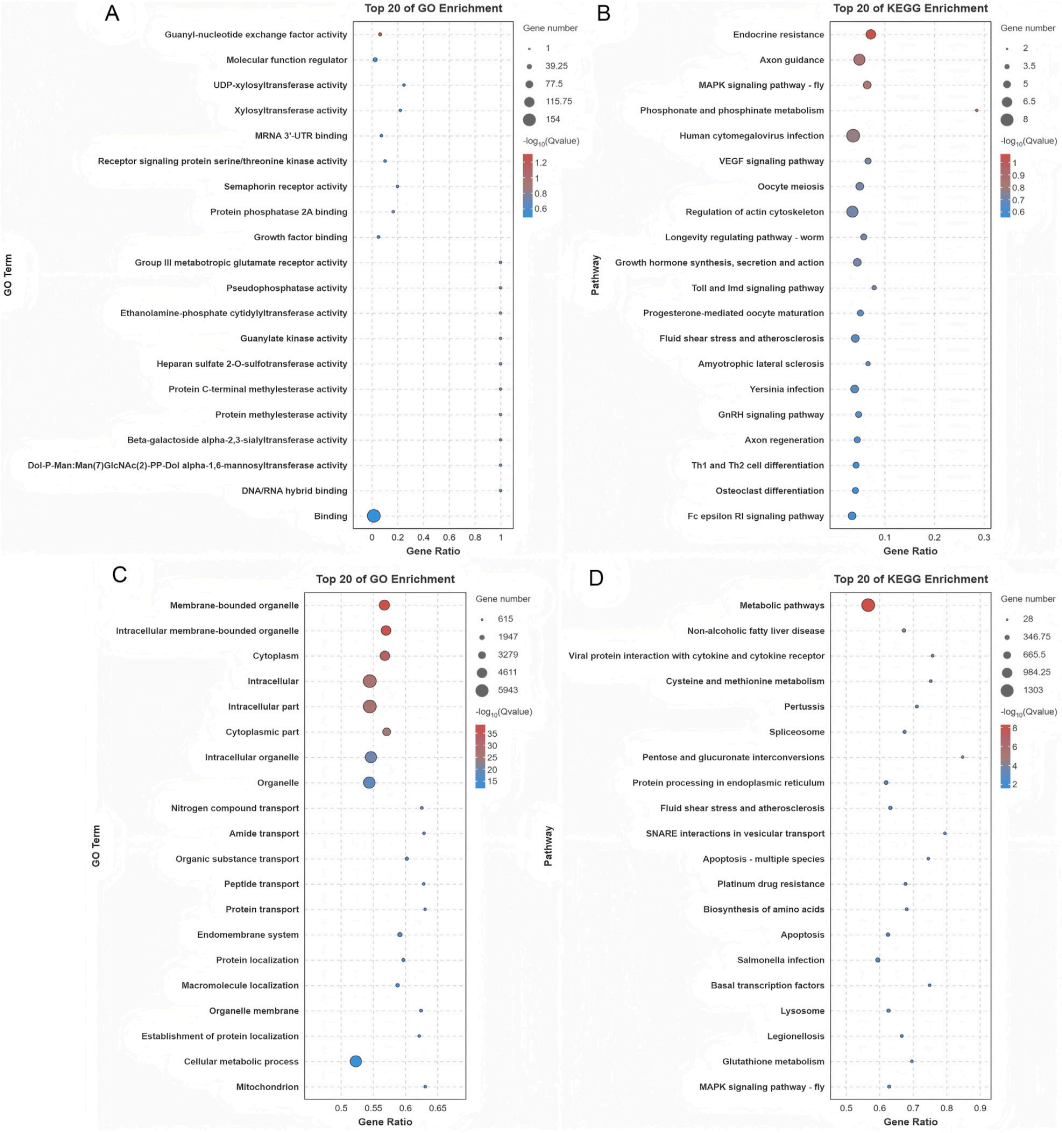
Functional enrichment analysis of genes associated with differentially expressed circRNAs and miRNAs. **(A)** GO enrichment analysis of genes originating from the parental sources of differentially expressed circRNAs. **(B)** KEGG pathway analysis of the parental genes of differentially expressed circRNAs. **(C)** GO enrichment analysis of genes regulated by differentially expressed miRNAs. **(D)** KEGG pathway analysis of genes targeted by differentially expressed miRNAs.

GO analysis of DE circRNAs identified 414 enriched terms (*p* < 0.05), linked to transcription, signaling, and transport (Figure 6A; Supplementary Table S7). KEGG analysis revealed 32 enriched pathways, including MAPK, VEGF, and GnRH signaling. These pathways play essential roles in regulating hormonal responses, cellular growth, and oocyte maturation, processes that are fundamental to reproductive biology. The top 20 pathways are presented in Figure 6B, while a comprehensive list of additional pathways can be found in Supplementary Table S7.

#### 3.3.4 DE miRNAs

Target genes for DE miRNAs were predicted, resulting in the identification of 580 unique target genes and 2,193 predicted binding sites across 90 DE miRNAs (Supplementary Table S8). Functional enrichment analysis was performed to investigate the biological significance of these genes. GO analysis revealed significant enrichment of 2,099 terms (*p* < 0.05), with a predominant association to key processes like protein transport and various metabolic pathways. These processes are crucial for maintaining cellular functionality and regulating molecular networks within cells (Figure 6C; Supplementary Table S8). Further analysis using KEGG pathway investigation highlighted that the target genes were strongly implicated in several vital biological pathways. Among these, the MAPK signaling, Hippo signaling, and AMPK signaling pathways were particularly prominent, all of which are involved in regulating essential cellular functions such as growth, energy balance, and cellular responses to stress. The 20 most enriched pathways are illustrated in Figure 6D, and a more detailed list of additional pathways can be found in Supplementary Table S8.

### 3.4 Construction of competing endogenous RNA (ceRNA) network

**FIGURE 7:**
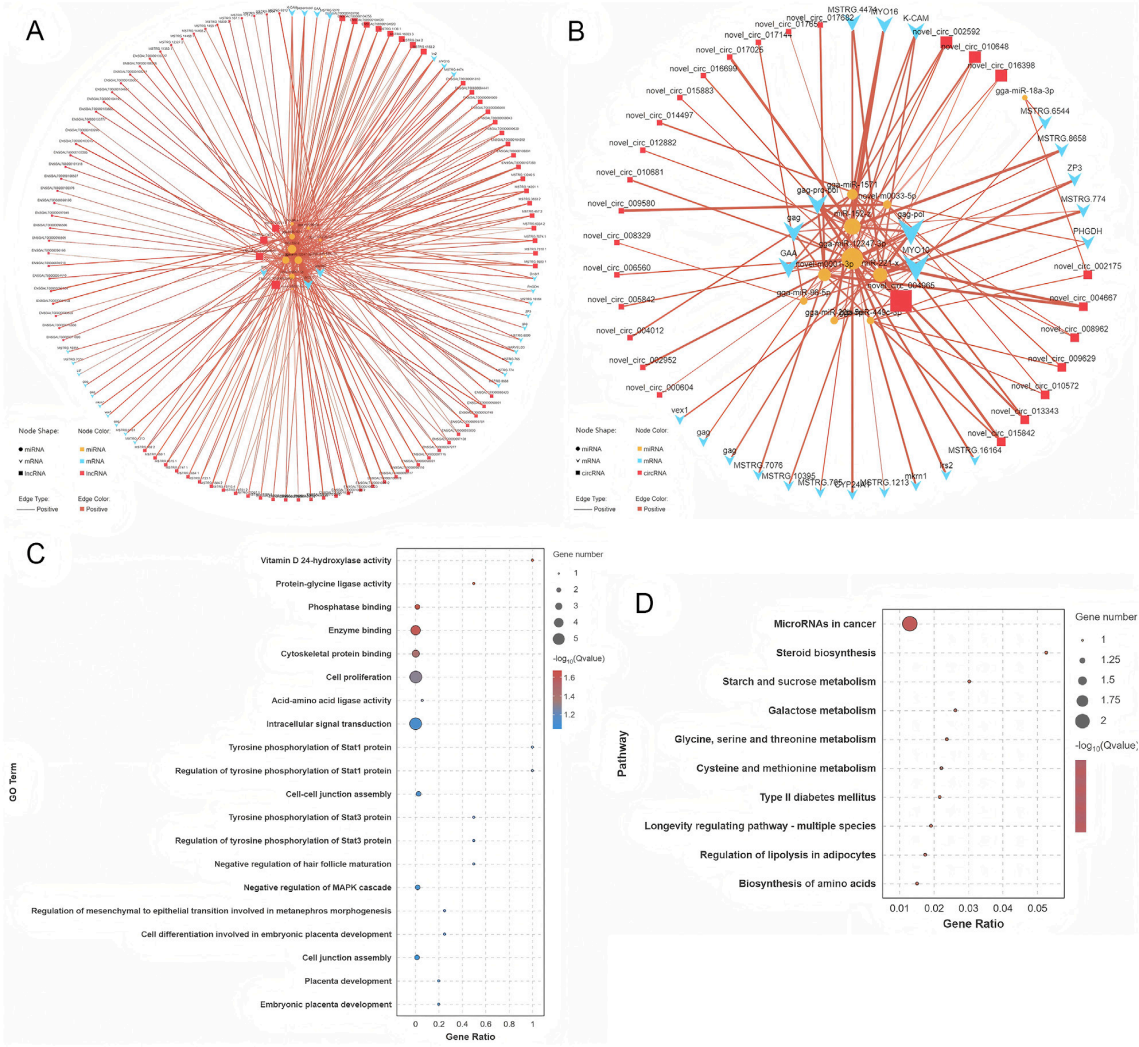
ceRNA network construction and functional analysis. **(A)** ceRNA network associated with circRNAs. **(B)** ceRNA network associated with lncRNAs. **(C)** GO functional enrichment analysis of the target mRNAs. **(D)** KEGG pathway analysis of the target mRNAs.

The target genes of DE miRNAs were initially predicted. This was followed by computation of expression correlations using the SCC. Target gene pairs with SCC < −0.7 were selected for further analysis (Supplementary Table S9). Next, PCC was used to compute expression correlations, and pairs with PCC > 0.9 were considered potential ceRNA interactions. To refine the ceRNA pairs, a statistical threshold with a *p*-value of less than 0.05 was applied, ultimately identifying 421 lncRNA-miRNA-mRNA pairs and 93 circRNA-miRNA-mRNA pairs (Supplementary Table S9). These selected ceRNA interactions were used to construct a ceRNA interaction network, which incorporates the expression patterns of lncRNA, circRNA, miRNA, and mRNA (Supplementary Figure S2). Within this network, RNA molecules with higher connectivity are likely to play more significant roles in the regulation of follicle selection, as they have stronger regulatory potential. The connectivity analysis revealed the top ten RNA molecules with the highest connectivity: MSTRG.244, MSTRG.244.2, MSTRG.5180, novel_circ_004965, MSTRG.6379, MSTRG.9372, MSTRG.1650, MSTRG.2966, MSTRG.6544, and MSTRG.4751.2 (Supplementary Figure S3). These RNAs are promising candidates for further investigation regarding their role in the regulation of follicle selection in Luxi gamecock. Several miRNAs, including miR-12247-3p, are key regulators in the network (Figures 7A,B).

GO analyses of ceRNA network mRNAs revealed enrichment of cell proliferation, signaling, and metabolic pathways (Figure 7C). KEGG pathway analyses identified significantly enriched pathways, including MicroRNAs in cancer, steroid biosynthesis, starch and sucrose metabolism, and galactose metabolism (Figure 7D). These pathways are involved in a range of cellular activities, including metabolic processes, hormonal regulation, and the molecular mechanisms underlying cancer progression, offering valuable insights into the biological roles of the ceRNA network.

**FIGURE 8:**
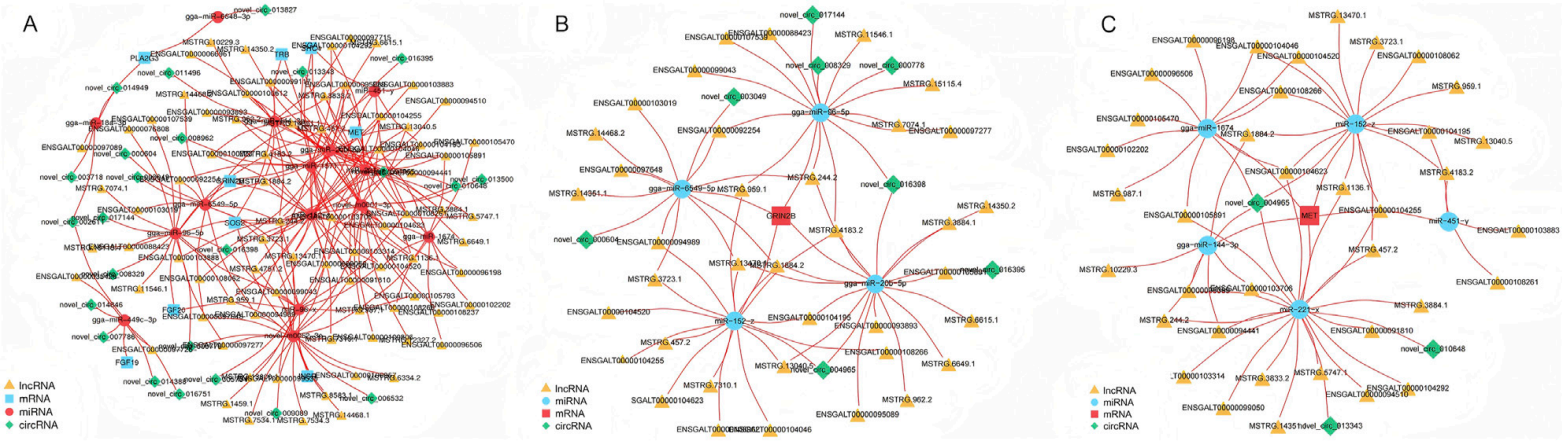
Visualization of crucial genes in the ceRNA network. **(A)** The ceRNA subnetworks of differentially expressed mRNAs in Ras signaling pathway. **(B)** The ceRNA subnetwork of GRIN2B. **(C)** The ceRNA subnetwork of MET.

In our previous KEGG pathway analysis, significant enrichment was observed in the Ras signaling pathway. To further explore the interactions between mRNAs, lncRNAs, circRNAs, and miRNAs within this pathway, a ceRNA network was constructed. This network aims to uncover the complex relationships among these RNA molecules specifically in the context of the Ras signaling pathway. The constructed ceRNA regulatory network comprises 43 circRNA-miRNA-mRNA interaction pairs and 227 lncRNA-miRNA-mRNA interaction pairs (Figure 8A; Supplementary Table S10). For instance, the expression of the GRIN2B gene was found to be regulated by 37 distinct lncRNAs, with a subnetwork involving eight circRNAs and four miRNAs (gga-miR-20b-5p, gga-miR-96-5p, gga-miR-6549-5p, miR-152-z) that might influence its expression (Figure 8B). Similarly, the MET gene, known for its involvement in numerous cellular processes, is regulated by 38 different lncRNAs. Its expression is potentially influenced by a subnetwork containing three circRNAs and five miRNAs (miR-221-x, gga-miR-144-3p, miR-152-z, gga-miR-1674, miR451-y) (Figure 8C). These interactions within the ceRNA network provide a more in-depth understanding of the molecular mechanisms regulating the Ras signaling pathway.

## 4 Discussion

Luxi gamecock is a precious local breed with limited egg production. The process of egg laying is biologically complex and highly regulated, involving an intricate network of genes, both coding and non-coding RNAs, as well as various signaling pathways that coordinate this process (Guo et al., 2019). The selection and development of follicles is a key factor influencing egg production levels (Johnson, 2015). To gain insight into the molecular mechanisms behind follicle selection and to further enhance egg production in Luxi gamecock, comprehensive transcriptome sequencing of both SYFs and LYFs was conducted. This analysis led to the identification of 1,113 DE mRNAs, 245 DE lncRNAs, 264 DE circRNAs, and 90 DE miRNAs. Some of these DE mRNAs and DE miRNAs were subsequently validated by qRT-PCR, and the results showed a high degree of consistency with the RNA-seq data.

A set of genes already known to play a role in follicle selection was identified among the DE mRNAs. For instance, AMH (Anti-Müllerian Hormone), a growth and differentiation factor in the TGF-β superfamily, is highly expressed in smaller follicles before follicle selection begins. However, its expression decreases significantly during the selection phase (Johnson et al., 2008). When AMH is present in excess, it can inhibit follicle selection, making the proper regulation of AMH expression crucial for this process, as well as for improving reproductive efficiency (Johnson et al., 2009). Furthermore, the expression levels of STAR (steroidogenic acute regulatory protein) and CYP11A1 (cytochrome P450 family 11 subfamily A member 1), which are essential for the synthesis of progesterone, are closely linked with follicle selection (Johnson et al., 2002). GDF9 (growth differentiation factor 9) plays a crucial role in the formation of follicles and the proliferation of granulosa cells in chickens, which in turn promotes follicle selection and subsequent development. A lack of GDF9 can impair follicle growth, potentially leading to infertility (Hlokoe et al., 2022). Transcriptome and proteome analyses of the SYFs and selected follicles revealed significant enrichment of differential genes, such as VLDLR (very low density lipoprotein receptor), WIF1 (WNT inhibitory factor 1), and AMH, with AMH showing a marked reduction in the selected follicles (Chen et al., 2020). These findings further confirm the reliability and accuracy of the data obtained in this study. Functional analyses of DE mRNAs indicated that many of them were enriched in processes associated with organism development and morphogenesis, such as cell differentiation, tissue development, single-multicellular organism process, organ morphogenesis, single-organism developmental process, and development process. Genes such as VLDLR, WIF1, BMP15 (bone morphogenetic protein 15), and EGF (epidermal growth factor), which have been proven to influence follicle selection in chickens, were identified as being involved in several of these processes (Chen et al., 2020; Stephens and Johnson, 2016; Johnson et al., 2015; Lin et al., 2011; Nie et al., 2024). Moreover, numerous signaling pathways were significantly enriched, including the Ras signaling pathway, ovarian steroidogenesis, and Notch signaling pathway. The Ras signaling pathway, known to be a central mechanism within cells, regulates various biological functions, such as cell growth, differentiation, apoptosis, and metabolism (Sadeghi Shaker et al., 2023). This pathway interacts with other key pathways like MAPK, PIP3, and Rac/Rho signaling, creating a complex network of signal transduction mechanisms (Huang et al., 2023). While the role of the Ras signaling pathway in follicle selection remains unclear, the involvement of MAPK and PIP3 pathways in chicken follicle selection has been established (Woods et al., 2007). Studies have shown that the MAPK signaling pathway may be involved in regulating follicle selection (Xu et al., 2018). Therefore, it is plausible that Ras signaling may also contribute to this process, though its exact mechanism requires further investigation. Ovarian steroidogenesis is another critical process in follicle selection. In this study, BMP15 and CYP11A1 were found to be significantly enriched in this pathway. BMP15 was downregulated and CYP11A1 was upregulated in LYFs, consistent with findings from recent studies (Stephens and Johnson, 2016; Johnson et al., 2002). Notch signaling pathway can regulate follicle development and estradiol production (Jing et al., 2017; Chen et al., 2014). Additionally, GSEA further identified key gene sets with differential expression between SYFs and LYFs. The analysis highlighted pathways associated with thyroid hormone synthesis, Notch signaling, AMPK signaling, and Hippo signaling, all of which are integral to follicle development (Jing et al., 2017; Hu et al., 2023; Lv et al., 2019). The Hippo signaling pathway is involved in chicken follicle selection (Sun et al., 2021; Lyu et al., 2016). These findings suggest that DE mRNAs may regulate follicle selection in Luxi gamecock by influencing these processes, and cross-talk between the key signaling pathways may occur at the level of key regulatory molecules. These molecules could act as “hubs” for integrating signals from different pathways, thereby precisely regulating follicle selection; however, the precise molecular mechanisms remain to be explored.

LncRNAs regulate gene expression through cis and trans mechanisms (Liu et al., 2019; Isales et al., 2020), influencing various biological processes (Weikard et al., 2017; Quan et al., 2015). The construction of regulatory networks represents the most effective approach to understanding the pathways where lncRNAs function (Chen R. et al., 2018). Co-expression network analysis showed DE lncRNAs targeting DE mRNAs enriched in key pathways. These pathways include steroid hormone biosynthesis, Ras signaling, ovarian steroidogenesis, and the Notch signaling pathway. Previous studies have consistently demonstrated that lncRNAs can regulate their target genes and subsequently participate in the regulation of complex processes such as follicle growth and development, gonad development, and hormonal regulation (Statello et al., 2020). In this study, 245 DE lncRNAs were identified. Among them, target genes such as NR5A2, CYP11A1, and GDF9, which were targeted by specific lncRNAs (ENSGALG00000002182, ENSGALG00000034982, ENSGALG00000028407) are closely linked to follicle selection (Guo et al., 2022; Johnson et al., 2002; Hlokoe et al., 2022). These differentially expressed lncRNAs are likely to serve as important regulators of the follicle selection process in Luxi gamecock. Through their interactions with specific target genes, these lncRNAs could modulate critical pathways involved in follicle development and reproductive function. The identification of these lncRNAs lays the groundwork for further exploration into their roles and regulatory mechanisms in poultry reproduction.

CircRNAs play important roles in a wide array of biological functions, including but not limited to cell proliferation, signal transduction, and maintaining genomic stability (Greene et al., 2017). A total of 264 DE circRNAs were identified, with parental genes enriched in MAPK, VEGF, progesterone-mediated oocyte maturation, and GnRH pathways. This is similar to the results of other studies on follicle development (Liu et al., 2024a; Wang et al., 2024; Liu et al., 2024b). CircRNA expression in granulosa cells from SYFs showed enrichment in ovarian steroidogenesis, MAPK, and PI3K/Akt pathways (Wang et al., 2022). For example, CircRPS19 has been shown to regulate granulosa cell development by alleviating the inhibitory effects of miR-218-5p on INHBB (inhibin beta B subunit), which suggests a novel regulatory mechanism involving both circRNA and miRNA in the development of ovarian follicles in chickens (Wei et al., 2024). Given that circRNAs exert their biological effects through various molecular mechanisms, further research is necessary to elucidate their precise role and the underlying mechanisms in follicle selection and other related reproductive processes.

miRNAs regulate follicle development, atresia, proliferation, apoptosis, and steroid biosynthesis (Wang et al., 2021; Xu et al., 2024; He et al., 2022; Wei et al., 2022). In the current study, a total of 90 DE miRNAs were identified, targeting 580 genes and 2,193 predicted binding sites. These miRNAs are implicated in a variety of cellular processes. For example, miR-31 has been shown to regulate apoptosis in pig ovarian granulosa cells during follicle growth. It achieves this by targeting the FSHR and HSD17B14 genes, resulting in a reduction in P4 levels within these cells (Du et al., 2020). Sequencing the granulosa cells of follicles at different developmental stages showed significant differences in miR-449c-3p expression. These differences are associated with the MAPK, TGF-β, and Wnt signaling pathways, which are associated with follicle selection, indicating that miRNA may be involved in regulating this process (Shen et al., 2023). miR-128-3p promoted granulosa cell apoptosis and reduced the secretion of progesterone and estrogen; thus, it may also be associated with follicle selection (Ning et al., 2023). DE miRNAs target genes enriched in AMPK, ovarian steroidogenesis, and Hippo pathways, regulating follicle selection. The involvement of these pathways in reproductive functions, particularly in regulating cellular differentiation and hormone synthesis, highlights the critical role miRNAs might have in controlling the complex processes of follicle development and selection (Johnson et al., 2002; Hu et al., 2023; Sun et al., 2021). Therefore, understanding how these miRNAs interact with specific signaling pathways could provide deeper insights into their role in reproductive biology. These results demonstrate how DE miRNAs might participate in regulating follicle selection by altering gene expression in these pathways. Additionally, an intriguing phenomenon was observed that DE miRNAs exhibited a differential expression pattern distinct from that of other RNAs. The expression of DE miRNAs was influenced by individual samples, indicating that there might be inter-individual heterogeneity in miRNA regulatory mechanisms of Luxi gamecocks. These findings implied that miRNAs may modulate follicle selection through network-level interactions, a role potentially different from that of other RNAs. This has provided a new research direction for future study, for example, to employ population-level miRNA sequencing to quantify the association between individual miRNA variability and follicle selection, thereby elucidating their regulatory networks.

The ceRNA mechanism plays a crucial role in biological and pathological processes (Singh et al., 2022). The ceRNA network that regulates follicle development in livestock is well documented. Bovine granulosa cell transcriptome analysis identified lncRNAs involved in cystic follicle formation (Wang K. et al., 2022). Similarly, the construction of a ceRNA network for buffalo follicles, based on whole-transcriptome analysis, revealed the regulatory roles of DE RNAs in granulosa cells, where lncRNAs were found to interact dynamically with target genes in a ceRNA fashion. This interaction underscores their significant role in buffalo follicular development and atresia (Pan et al., 2021). Additionally, experiments targeting ciRS-187 in bovine cumulus cells showed that knocking down ciRS-187 upregulated the expression of BMPR2 (bone morphogenetic protein receptor type 2), further supporting the involvement of circRNAs in the regulation of follicle development through a ceRNA mechanism (Fu et al., 2023). CeRNA networks regulate ovarian function in poultry, with key miRNAs identified in goose follicle selection (Liu et al., 2023). These studies collectively demonstrate the crucial role of ceRNA networks in follicular development. Building upon this, the present study identified several ceRNA networks that are potentially involved in regulating follicle selection. Among them, novel_circ_009629-miR-12273-5p-PHGDH and ENSGALT00000090691-miR-12273-5p-PHGDH were identified as potential regulatory interactions. PHGDH (phosphoglycerate dehydrogenase), a key enzyme in the serine biosynthesis pathway, is a known rate-limiting enzyme and has been associated with the growth and progression of various cancers (Lee et al., 2024). However, its role in chicken follicle selection remains unclear and warrants further investigation. Additionally, CYP24A1 (cytochrome P450 family 24 subfamily A member 1) was also identified as a key gene in a ceRNA network, with potential regulatory interactions like novel_circ_004965-miR-144-3p-CYP24A1 and MSTRG.10229.3-miR-144-3p-CYP24A1, which could also be involved in follicle selection. This study uncovered multiple ceRNA networks, highlighting the possibility that these genes may influence follicle selection through complex ceRNA regulatory mechanisms.

To further clarify the regulatory role of key signaling pathways in follicle selection, the significantly enriched Ras signaling pathway was selected to construct the ceRNA regulatory network of DE mRNAs involved in it. The Ras signaling pathway is involved in many biological processes, such as cell growth, differentiation, apoptosis, and metabolism (Sadeghi Shaker et al., 2023), and it can interact with MAPK, PIP3, and Rac/Rho pathways (Huang et al., 2023). A ceRNA network identified GRIN2B (glutamate ionotropic receptor NMDA type subunit 2B) and MET (MET proto-oncogene, receptor tyrosine kinase) as key genes regulated by lncRNAs, circRNAs, and miRNAs. GRIN2B plays an important role in brain development, circuit formation, and perhaps cellular migration and differentiation, as well as synaptic plasticity (Hu et al., 2016). However, to date, there is no research linking GRIN2B to animal reproduction, making it an intriguing target for future studies in reproductive biology. On the other hand, MET is a proto-oncogene that has been extensively studied for its role in cancer. It is crucial for regulating cell growth, proliferation, and differentiation (Shirasaki et al., 2022). However, its potential involvement in reproductive functions remains unexplored, and it represents another promising avenue for further investigation. Based on these findings, the author hypothesized that follicle selection in Luxi gamecock might be influenced by key signaling pathways that are regulated through ceRNA networks (lncRNA/circRNA–miRNA–mRNA). The exact molecular mechanisms underlying these interactions require further investigation. Furthermore, GRINB and MET should be studied as potential molecular markers to clarify their roles in regulating follicle selection, which could improve the egg-laying performance of Luxi gamecock.

This study also had certain limitations. Firstly, due to the difficulties in obtaining follicle samples from Luxi gamecocks, the sample size may be relatively limited. This may have restricted the power of the statistical analyses and made it difficult to capture all the differential characteristics within the Luxi gamecocks. Secondly, although high-throughput sequencing technology was employed, some low-abundance transcripts might be missed in detection. Some rare RNAs or those with extremely low expression levels might have been overlooked, which could affect understanding of the complete regulatory network involved in follicle selection. Additionally, this study has limitations in the depth of result mining. Some potential regulatory mechanisms remain insufficiently elucidated, and the action mechanisms of non-additive effects, which might influence follicle selection, still need to be further explored. Subsequent studies should increase the sample size, optimize the sequencing strategy and analysis methods, and deepen data mining and analysis to further advance the research on the follicle selection mechanism in Luxi gamecocks.

## 5 Conclusion

In summary, mRNA, lncRNA, circRNA, and miRNA transcript profiles of the SYFs and LYFs were characterized using RNA-seq, leading to the identification of DE mRNAs, DE lncRNAs, DE circRNAs, and DE miRNAs associated with follicle selection. Further functional analysis highlighted that many of these DE RNAs are actively involved in biological processes central to follicle selection. ceRNA regulatory networks were constructed to explore the molecular regulatory mechanisms. Several key RNAs, GRIN2B and MET, have not been previously implicated in follicle selection. This novel discovery may offer fresh perspectives for further exploring the mechanisms of follicle selection in chickens, and can also be studied as potential molecular markers for further research to improve the egg-laying performance of Luxi gamecock. Overall, the findings of this study enhance the comprehension of the molecular processes involved in follicle selection and could provide foundational knowledge to support the improvement in egg-laying performance in Luxi gamecock. Subsequent studies will investigate the functions of the key RNAs in regulating follicle selection, compare expression levels between high and low egg production individuals within the Luxi gamecock breed, and explore the specific regulatory mechanisms within the ceRNA network to further validate the potential of the identified RNAs as molecular markers and gain a deeper understanding of the molecular mechanisms influencing egg-laying performance.

## Data Availability

The raw datasets for this study can be found in the National Center for Biotechnology Information (NCBI) Sequence Read Archive (SRA), with accession number PRJNA1214885.
