# Supplementary figures and images for: Whole-transcriptome insights into follicle selection: deciphering key regulatory networks in Luxi gamecock
Source: Front Genet. 2025 Aug 6;16:1620058. doi: 10.3389/fgene.2025.1620058 (PMC12364954; doi:10.3389/fgene.2025.1620058)

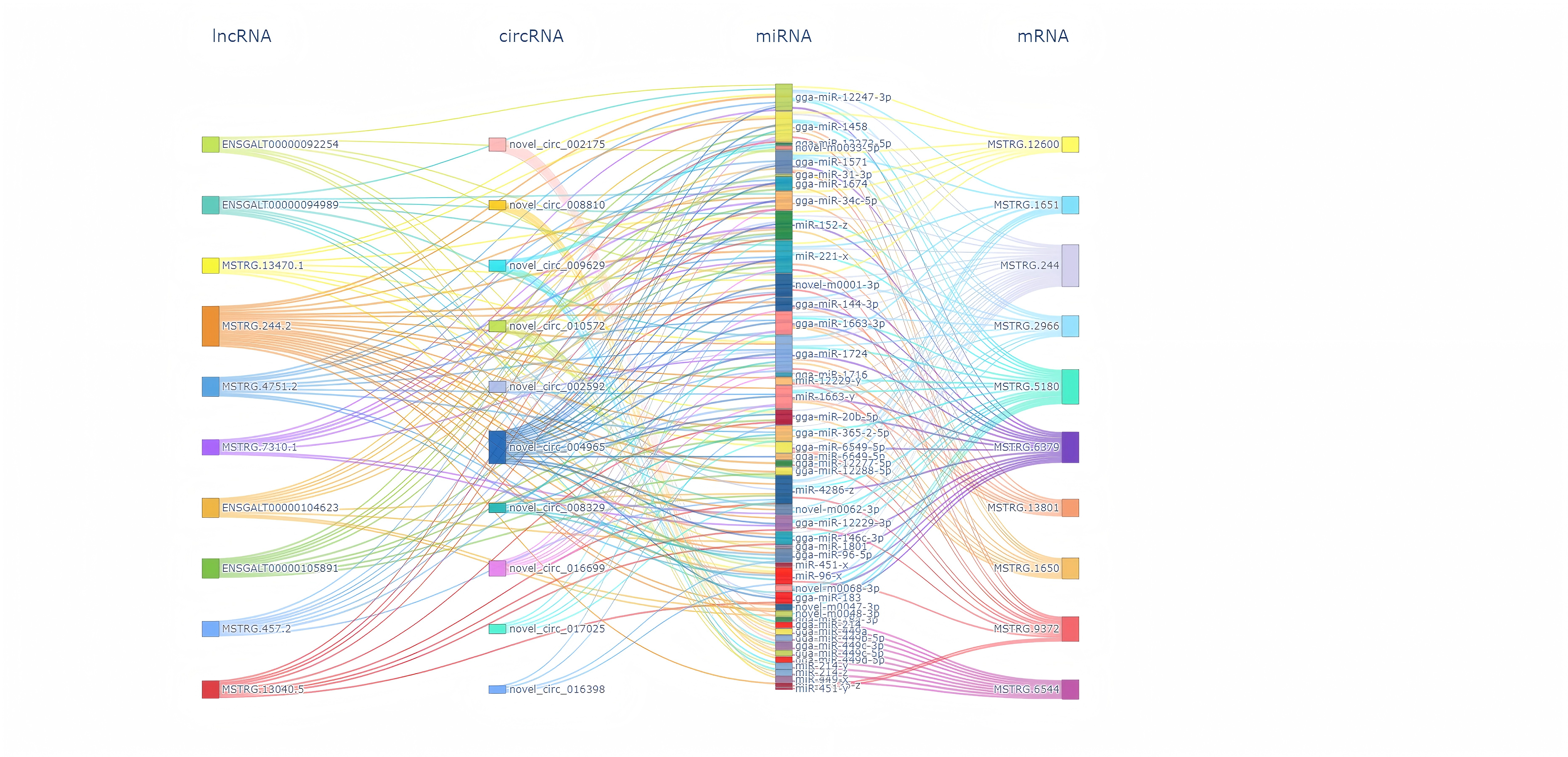

Supplement: Supplementary file 2 [file Image3.jpeg]

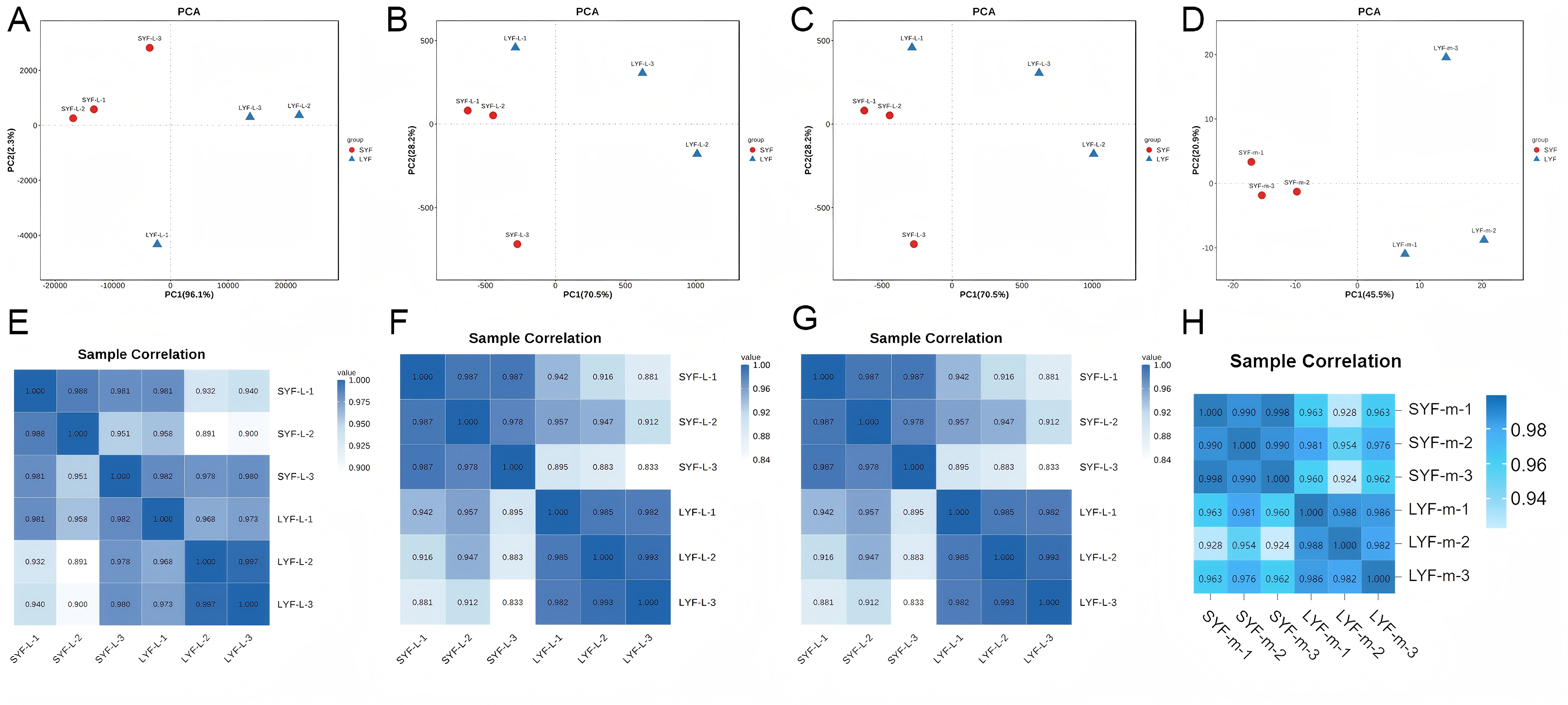

Supplement: Supplementary file 5 [file Image1.jpeg]

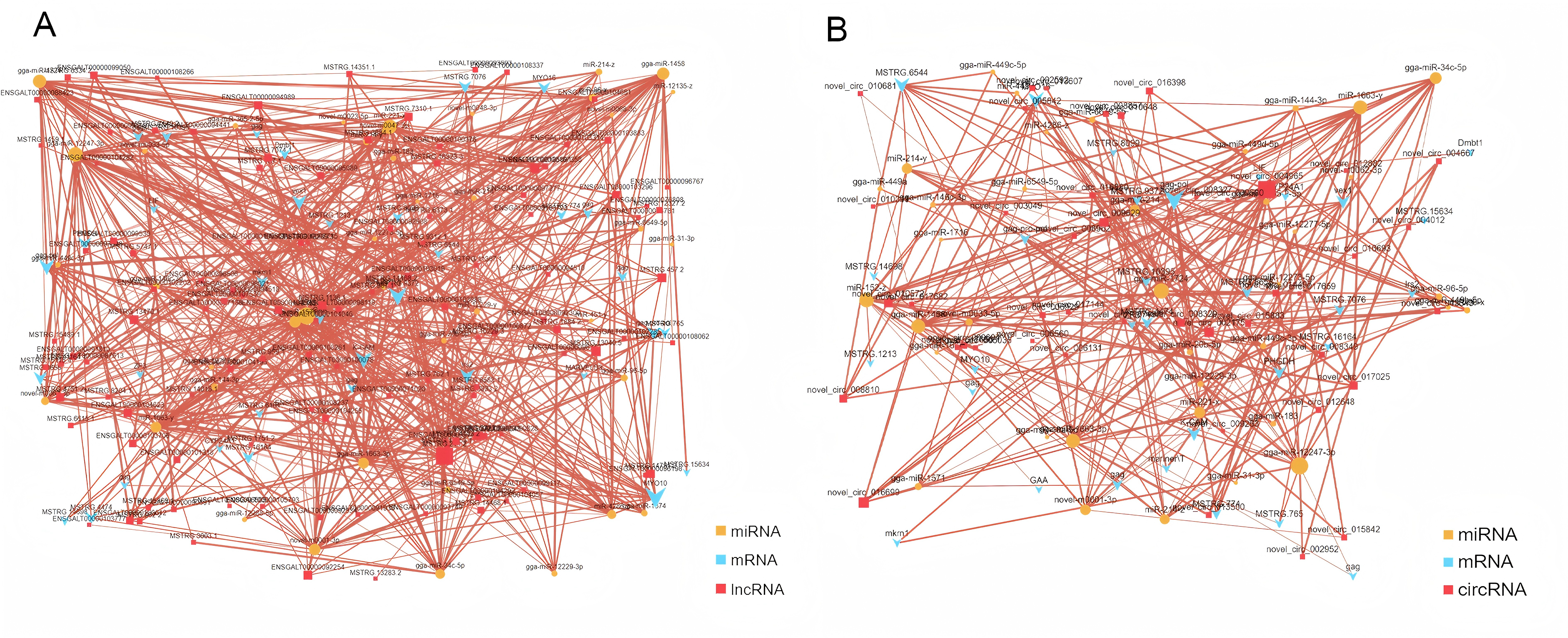

Supplement: Supplementary file 6 [file Image2.jpeg]
