# Supplementary material for: Whole-transcriptome insights into follicle selection: deciphering key regulatory networks in Luxi gamecock
Source: Front Genet. 2025 Aug 6;16:1620058. doi: 10.3389/fgene.2025.1620058 (PMC12364954; doi:10.3389/fgene.2025.1620058)
Supplement: Supplementary file 14 [file DataSheet1.docx]

Supplementary Material

# Supplementary Figures and Tables

## Supplementary Figures


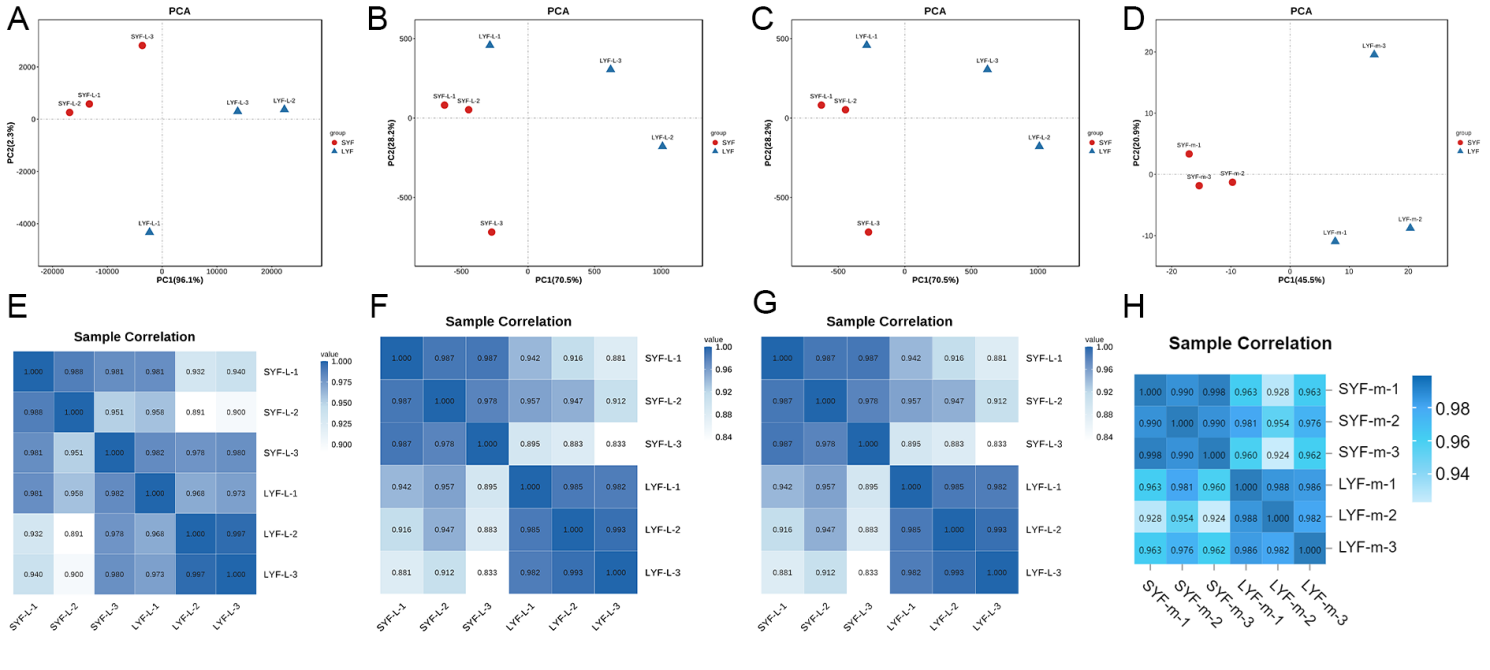


**Supplementary Figure 1.** PCA and heatmap analysis of sample correlations. (A) PCA of mRNA expression profiles across different samples. (B) PCA of lncRNA expression profiles across different samples. (C) PCA of circRNA expression profiles across different samples. (D) PCA of miRNA expression profiles across different samples. The heatmap represents the differential expression analysis of (E) mRNAs, (F) lncRNAs, (G) circRNAs, and (H) miRNAs. Correlation between sample expression profiles was determined using Pearson’s correlation coefficient based on the total transcript expression levels.


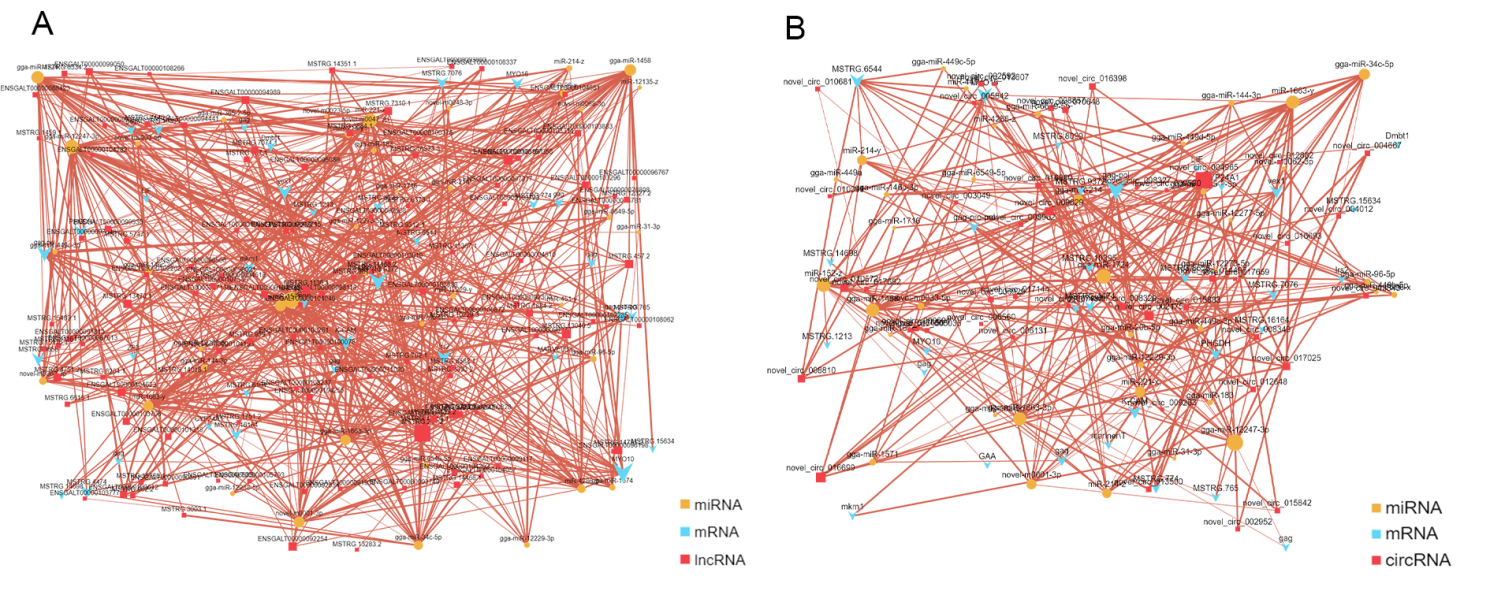


**Supplementary Figure 2.** Visualization of the ceRNA networks. (A) lncRNA-miRNA-mRNA network. (B) circRNA-miRNA-mRNA network.


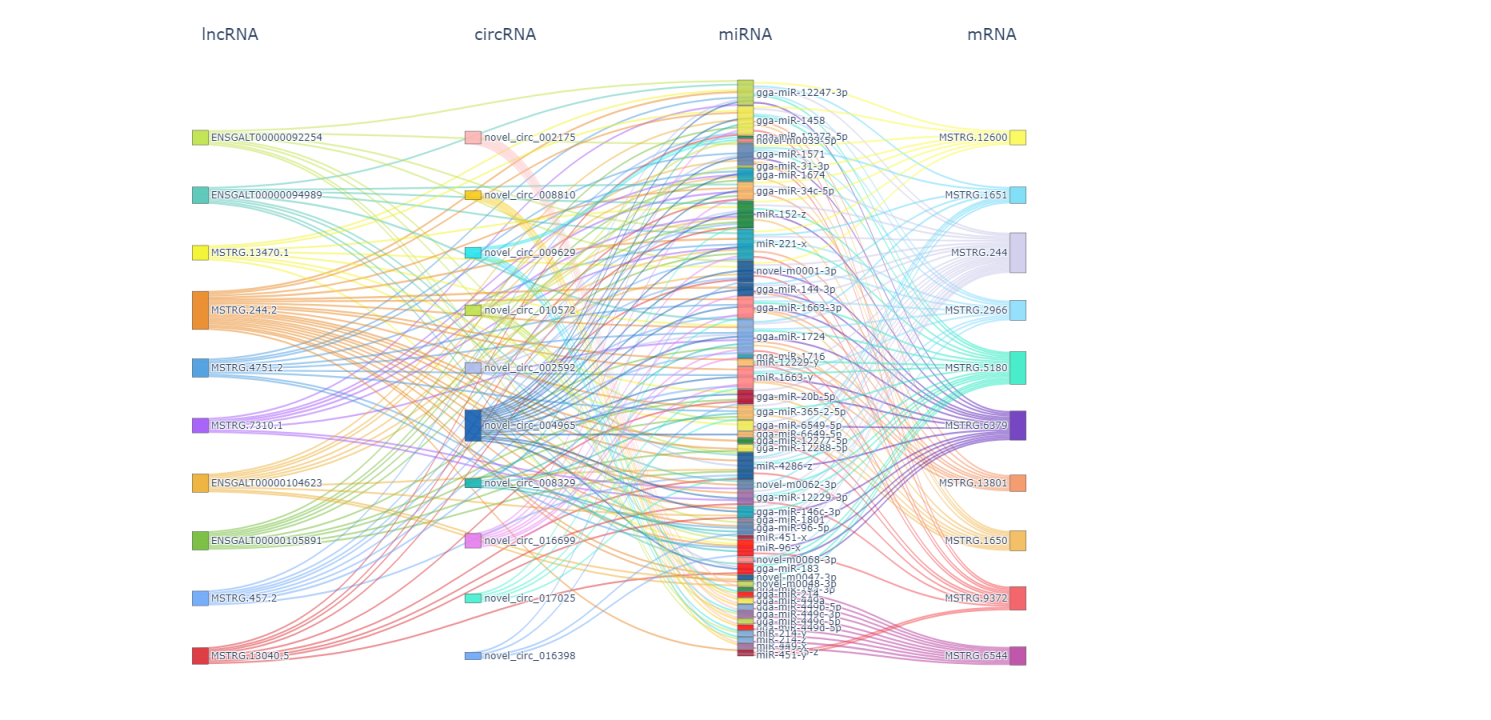


**Supplementary Figure 3.** The top ten molecules with the highest connectivity in the ceRNA network.

## Supplementary Tables

**Supplementary Table 1.** Primers used for qRT-PCR.

**Supplementary Table 2.** Summary of the RNA-seq results.

**Supplementary Table 3.** All expressed mRNAs, lncRNAs, circRNAs and miRNAs.

**Supplementary Table 4.** Differentially expressed mRNAs, lncRNAs, circRNAs and miRNAs.

**Supplementary Table 5.** Functional analysis of DE mRNAs.

**Supplementary Table 6.** Functional analysis of DE lncRNAs.

**Supplementary Table 7.** Functional analysis of DE circRNAs.

**Supplementary Table 8.** Functional analysis of DE miRNAs.

**Supplementary Table 9.** Construction of ceRNA.

**Supplementary Table 10.** ceRNA network of DEGs involved in Ras signaling pathway.

# Data availability statement

The raw datasets for this study can be found in the National Center for Biotechnology Information (NCBI) Sequence Read Archive (SRA), with accession number PRJNA1214885.
